# Prevalence, incidence and risk factors of visual disability in patients with exudative age-related macular degeneration: a nationwide population-based study in Korea

**DOI:** 10.3389/fmed.2026.1766076

**Published:** 2026-03-05

**Authors:** Min Seok Kim, Seonghee Nam, Jeongwoo Lee, Se Joon Woo

**Affiliations:** 1 Department of Ophthalmology, Seoul National University College of Medicine, Seoul National University Bundang Hospital, Seongnam, Republic of Korea; 2 Data Research, Samil Pharm Co., Ltd., Seoul, Republic of Korea

**Keywords:** age related macular degeneration, exudative age related macular degeneration, Korea, population study, prevalence, blindness, visual disability

## Abstract

**Background:**

Age-related macular degeneration (AMD) is a leading cause of irreversible visual loss in the elderly. Although anti-vascular endothelial growth factor (VEGF) therapy has improved the visual prognosis of exudative AMD, a considerable proportion of patients still develop severe vision loss. However, real-world data on the prevalence, incidence and risk factors for visual disability among patients with exudative AMD remain limited. This study investigates the prevalence, incidence and risk factors for visual disability among patients with exudative AMD.

**Methods:**

This nationwide, population-based retrospective cohort study used Korean National Health Insurance Service data from 2009 to 2023. Patients with newly diagnosed exudative AMD were identified, with a two-year washout period (2009–2010). Prevalence, incidence, hazard ratio (HR) for visual disability were analyzed.

**Results:**

A total of 147,406 patients with exudative AMD were included. The prevalence of visual disability in 2023 was 4.82% and the overall incidence rate was 12.18 per 1,000 person-years. At 8 years, the cumulative incidence probability of monocular visual disability was 4.8% (95% CI, 4.6–5.1), and that of binocular visual disability was 4.4% (95% CI, 4.1–4.7). The mean duration from exudative AMD diagnosis to visual disability was 3.3 ± 2.6 years. The risk of visual disability increased with older age group (HR, 1.38; 95% CI, 1.35–1.42) and lower income level (HR, 1.06; 95% CI, 1.05–1.08). Female sex (HR, 1.41; 95% CI, 1.35–1.47), diabetic retinopathy (HR, 1.16; 95% CI, 1.08–1.25), glaucoma (HR, 1.10; 95% CI, 1.05–1.15), and severe intraocular hemorrhage requiring vitrectomy (HR, 3.14; 95% CI, 2.63–3.75) were also significant risk factors. A decreasing trend in visual disability incidence was observed among patients who were more recently diagnosed with exudative AMD (−0.25% point per year; *p* < 0.001).

**Conclusion:**

The burden of AMD-related visual disability remains significant, highlighting the need for strategies to improve treatment adherence and ensure equitable access to vision-preserving care. The decreasing trend of visual disability in recent years suggests the practical benefit of improved access to anti-VEGF treatment through lower drug costs and expanded insurance coverage.

## Introduction

Age-related macular degeneration (AMD) is a leading cause of irreversible visual loss among the elderly worldwide (1). With the advent of anti-vascular endothelial growth factor (VEGF) therapy, the visual prognosis of exudative AMD has substantially improved; however, a considerable proportion of patients still experience severe visual decline over time (2, 3). The burden of visual disability due to AMD extends beyond individual vision loss, influencing quality of life, healthcare utilization, and socioeconomic costs (4–7).

In South Korea, the National Health Insurance Service (NHIS) provides a unique opportunity to assess real-world evidence in a nationwide population, including data on visual disability certification (8). Although previous studies have focused on treatment efficacy and recurrence patterns in exudative AMD, limited evidence exists regarding the prevalence, incidence and risk factors of visual disability. Understanding when and in whom visual disability develops after an exudative AMD diagnosis is crucial for guiding patient counseling, healthcare resource allocation, and policy decisions related to visual disability benefits.

Therefore, this study aimed to investigate the prevalence, incidence and risk factors for visual disability among patients newly diagnosed with exudative AMD using data from the Korean NHIS database.

## Methods

### Ethics statement

This study was approved by the Institutional Review Board of Seoul National University Bundang Hospital (Approval No. X-2511-1006-901). It adhered to the principles of the Declaration of Helsinki and was conducted in accordance with good clinical practice guidelines. The need for informed consent was waived owing to the retrospective design of the study and the use of de-identified data.

### Data source

We conducted a retrospective cohort study using data from the Korean NHIS. The Korean NHIS database is a comprehensive, population-based claims database that includes nearly the entire Korean population. It contains detailed information on demographics, diagnoses, procedures, prescriptions, and healthcare utilization from all medical institutions covered by the national insurance system (8). Because enrollment in the NHIS is mandatory for all Korean residents, this database provides a unique opportunity to conduct large-scale, real-world epidemiologic studies with nationwide representativeness.

### Study population

Patients with exudative AMD were identified using the special registration code (V201) between January 1, 2009, and December 31, 2023. To construct a newly diagnosed cohort, individuals with a diagnosis of exudative AMD during the two-year washout period (January 1, 2009–December 31, 2010) were excluded. In addition, patients who had been certified with visual disability before the diagnosis of exudative AMD, those lacking information on insurance premiums, or those with missing data on age or sex, as well as individuals younger than 40 years, were excluded.

### Outcome and variable definition

In this study, visual disability was defined according to the Korean National Disability Registration System (grades 1–6), which is based on best-corrected visual acuity and visual field in the better or worse eye (Supplementary Table 1). These thresholds approximately correspond to moderate-or-worse visual disability and blindness in the World Health Organization classification, and therefore do not include mild visual disability. The main analysis was conducted using the presence and absence of visual disability as the dependent variable during the period from 2011 to 2023. Additionally, using data available up to 2019, a subgroup analysis was conducted by classifying grades 1–5 as binocular visual disability and grades 6 as monocular visual disability.

### Statistical analysis

Annual prevalence of visual disability among patients with exudative AMD was calculated for each calendar year from 2011 to 2023. Incidence rates of visual disability were calculated as the number of incident cases divided by the total person-time at risk and were expressed per 1,000 person-years. We compared incidence rates between groups using incidence rate ratio (IRR), calculated using Wald statistics based on the log-transformed rate ratio, assuming a Poisson distribution of incident events. Time-to-event analyses were performed using Cox proportional hazards regression to estimate hazard ratios (HR) for certification of visual disability among patients with exudative AMD. Covariates included income level, hypertension, diabetes mellitus, diabetic retinopathy, glaucoma, and intraocular hemorrhage requiring vitrectomy (Supplementary Table 2). Insurance premiums by 20-quantile level, which are classified according to income level in Korea, were used as a variable for income level. In Korea, National Health Insurance premiums are stratified into 20 income-based quantiles according to household income and assets, serving as a proxy for socioeconomic status. Patients were followed from the date of exudative AMD diagnosis until the year of visual disability or the end of the study period, whichever occurred first. Kaplan–Meier survival curves were plotted to visualize the cumulative incidence probability of visual disability. To evaluate temporal trends in the risk of visual disability across diagnosis years, we fitted a linear mixed-effects model. The annual incidence probability of visual disability was used as the dependent variable. Follow-up year (years since exudative AMD diagnosis) and diagnosis year were included as fixed effects, and diagnosis year was additionally specified as a random intercept to allow for heterogeneity in baseline risk across diagnosis years. A linear term for diagnosis year was used to estimate the yearly change in the incidence probability of visual disability.

All statistical analyses were conducted using SAS version 9.4 (SAS Institute, Cary, NC, United States) and R version 4.0.3 (R Foundation for Statistical Computing, Vienna, Austria). A two-sided *p*-value <0.05 was considered statistically significant.

## Results

**Figure 1:**
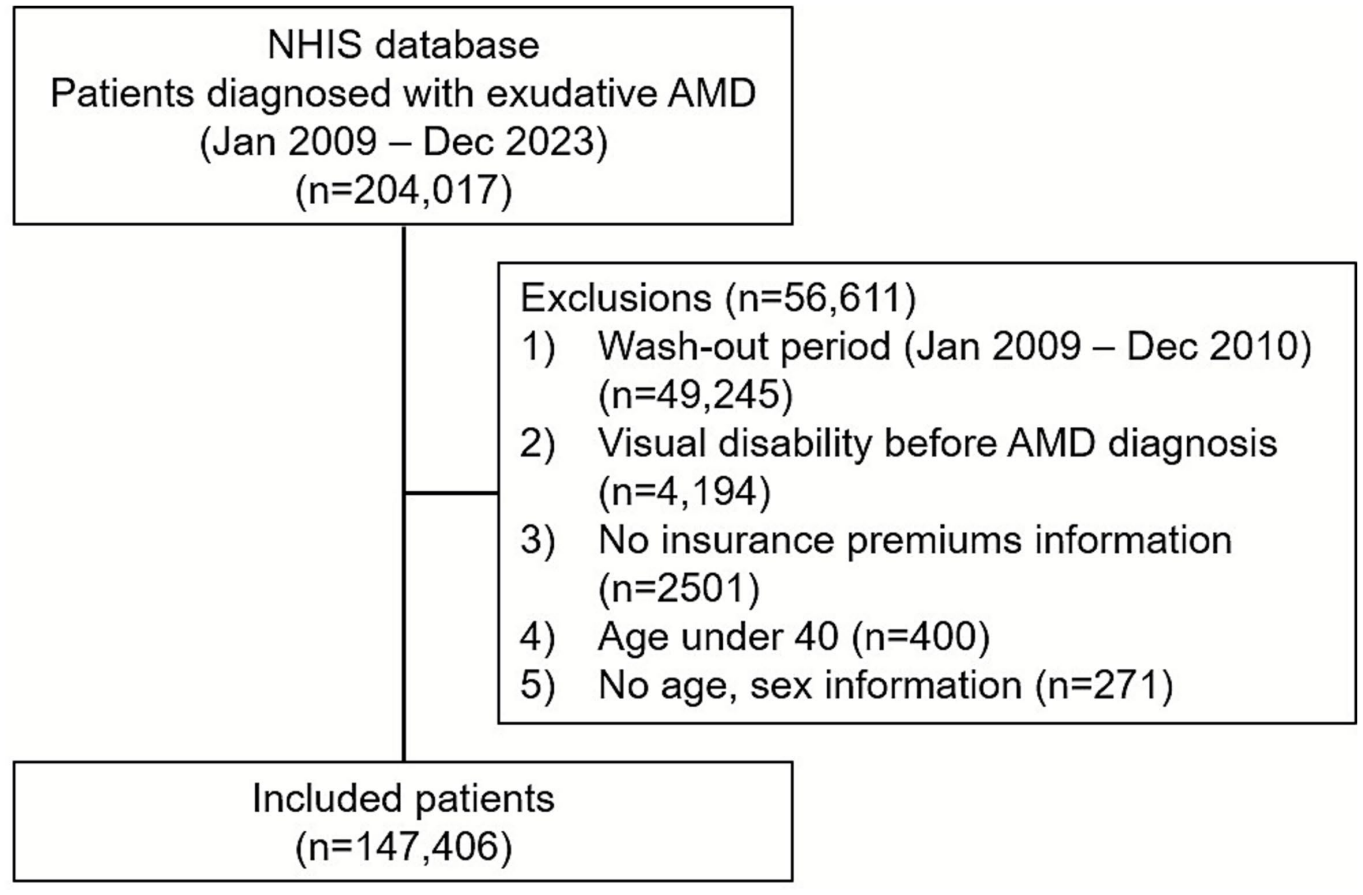
Flow diagram.

A total of 204,017 patients were diagnosed with exudative AMD between January 2009 and December 2023. After applying exclusion criteria, a total of 147,406 patients with exudative AMD were included in the analysis (Figure 1). The mean follow-up duration was 4.3 ± 3.3 years. The mean duration from the diagnosis of exudative AMD to visual disability certification was 3.3 ± 2.6 years.

Out of 145,406 exudative AMD patients, there were 7,537 (5.1%) patients with visual disability. The proportion of female sex was higher among patients with visual disability group compared to non-visual disability group (51.1% vs. 40.2%, *p* < 0.001). The mean age was 73.4 ± 9.8 years in female and 70.7 ± 9.5 years in male (*p* < 0.001), with median ages of 75 and 71 years, respectively. In addition, the prevalence of diabetic retinopathy, glaucoma, and intraocular hemorrhage requiring vitrectomy was higher in the visual disability groups (Table 1).

**Table 1:** Baseline characteristics.

Characteristics	Total	No visual disability	Visual disability	*p* ^c^	Binocular^a^	Monocular^a^	*p* ^c^
	(*n* = 147,406)	(*n* = 139,869)	(*n* = 7,537)		(*n* = 1,527)	(*n* = 2,095)	
Sex, *n* (%)							
Male	87,266 (59.2)	83,582 (59.8)	3,684 (48.9)	<0.001	671 (43.9)	1,139 (54.4)	<0.001
Female	60,140 (40.8)	56,287 (40.2)	3,853 (51.1)		856 (56.1)	956 (45.6)	
Age group, *n* (%)							
40–59	17,427 (11.8)	16,884 (12.1)	543 (7.2)	<0.001	71 (4.6)	197 (9.4)	<0.001
60–69	39,883 (27.1)	38,315 (27.4)	1,568 (20.8)		217 (14.2)	534 (25.5)	
70–79	55,958 (38.0)	52,460 (37.5)	3,498 (46.4)		746 (48.9)	976 (46.6)	
80~	34,138 (23.2)	32,210 (23.0)	1928 (25.6)		493 (32.3)	388 (18.5)	
Income level^b^							
Medical aid beneficiaries or Levels 1–3	26,100 (17.7)	24,604 (17.6)	1,496 (19.9)	<0.001	299 (19.6)	406 (19.4)	<0.001
Levels 4–7	14,982 (10.2)	14,202 (10.2)	780 (10.4)		152 (10)	237 (11.3)	
Levels 8–11	18,295 (12.4)	17,392 (12.4)	903 (12.0)		197 (12.9)	221 (10.5)	
Levels 12–15	25,292 (17.2)	24,058 (17.2)	1,234 (16.4)		257 (16.8)	348 (16.6)	
Levels 16–20	62,737 (42.6)	59,613 (42.6)	3,124 (41.5)		622 (40.7)	883 (42.1)	
Comorbidities							
Hypertension	87,908 (59.6)	83,342 (59.6)	4,566 (60.6)	0.086	398 (26.1)	466 (22.2)	0.004
Diabetes mellitus	35,424 (24.0)	33,550 (24.0)	1874 (24.9)	0.083	957 (62.7)	1,182 (56.4)	0.001
Diabetic retinopathy	18,460 (12.5)	17,324 (12.4)	1,136 (15.1)	<0.001	262 (17.2)	289 (13.8)	<0.001
Glaucoma	70,304 (47.7)	66,625 (47.6)	3,679 (48.8)	0.046	751 (49.2)	929 (44.3)	0.007
Intraocular hemorrhage requiring vitrectomy	819 (0.6)	694 (0.5)	125 (1.7)	<0.001	18 (1.2)	45 (2.1)	<0.001

a Data from the 2011–2019 dataset (*N* = 82,776).

b Classified by insurance premiums.

c Chi-square test.

In 2023, the prevalence of visual disability among patients with exudative AMD was 4.82% [95% confidence interval (CI), 4.70–4.94], corresponding to 6,092 cases of visual disability among 126,468 prevalent exudative AMD cases (Supplementary Table 3).

The overall incidence rate of visual disability among patients with exudative AMD was 12.18 cases per 1,000 person-years. Incidence rates were higher in female than in male (IRR, 1.52). Compared with adults aged 40–59 years, the incidence of visual disability increased progressively with age. Using medical aid beneficiaries or Levels 1–3 as the reference group, all higher-income groups demonstrated significantly lower incidence of visual disability (Table 2).

**Table 2:** Incidence rate of visual disability among exudative age-related macular degeneration.

Characteristics	Incidence rate, case/1,000 person-year	Incidence rate ratio (95% CI)	*p*
Overall	12.18		
Sex			
Male	10.04	Reference (1)	
Female	15.29	1.52 (1.45–1.59)	<0.001
Age group			
40–59	6.19	Reference (1)	
60–69	8.66	1.40 (1.27–1.54)	<0.001
70–79	14.65	2.37 (2.17–2.59)	<0.001
80~	17.29	2.79 (2.54–3.07)	<0.001
Income level			
Medical aid beneficiaries or Levels 1–3	15.14	Reference (1)	
Levels 4–7	11.80	0.78 (0.71–0.86)	<0.001
Levels 8–11	11.83	0.78 (0.72–0.85)	<0.001
Levels 12–15	11.36	0.75 (0.70–0.81)	<0.001
Levels 16–20	11.61	0.77 (0.72–0.83)	<0.001

Incidence rate ratios (95% CIs) were based on a Poisson model with Wald CIs.

In the Cox proportional hazards regression analysis, female sex (adjusted HR, 1.41), diabetic retinopathy (adjusted HR, 1.16), glaucoma (adjusted HR, 1.10), and intraocular hemorrhage requiring vitrectomy (adjusted HR, 3.14) were identified as significant risk factors for visual disability. The HR gradually increased with older age and lower income levels (Table 3). Trend analysis also revealed that higher age group (adjusted HR, 1.38; 95% CI, 1.35–1.42; *p* < 0.001) and lower income level (adjusted HR, 1.06; 95% CI, 1.05–1.08; *p* < 0.001) were significantly associated with a higher risk of visual disability.

**Table 3:** Cox proportional hazards model for the development of visual disability in patients with exudative age-related macular degeneration.

Characteristics	Univariable		Multivariable	
	Crude HR (95% CI)	*p*	Adjusted HR (95% CI)	*p*
Sex (reference: male)				
Female	1.52 (1.46, 1.59)	<0.001	1.41 (1.35, 1.47)	<0.001
Age group (reference: 40–59)				
60–69	1.39 (1.26, 1.53)	0.001	1.4 (1.27, 1.54)	<0.001
70–79	2.33 (2.13, 2.55)	<0.001	2.33 (2.12, 2.55)	<0.001
≥80	2.68 (2.43, 2.94)	<0.001	2.59 (2.34, 2.85)	<0.001
Income level^a^ (reference: Levels 16–20)				
Levels 12–15	0.98 (0.92, 1.04)	0.499	1.08 (1.01, 1.15)	0.033
Levels 8–11	1.02 (0.94, 1.09)	0.677	1.13 (1.05, 1.22)	0.001
Levels 4–7	1.02 (0.94, 1.10)	0.705	1.16 (1.08, 1.26)	<0.001
Medical aid beneficiaries or Levels 1–3	1.29 (1.21, 1.37)	<0.001	1.30 (1.22, 1.38)	<0.001
Comorbidities				
Diabetes mellitus	1.18 (1.12, 1.24)	<0.001	1.06 (1.00, 1.13)	0.052
Hypertension	1.13 (1.08, 1.18)	<0.001	0.92 (0.88, 0.97)	<0.001
Diabetic retinopathy	1.27 (1.19, 1.35)	<0.001	1.16 (1.08, 1.25)	<0.001
Glaucoma	1.16 (1.11, 1.21)	<0.001	1.10 (1.05, 1.15)	<0.001
Intraocular hemorrhage requiring vitrectomy	3.05 (2.56, 3.65)	<0.001	3.14 (2.63, 3.75)	<0.001

a Classified by insurance premiums.

**Figure 2:**
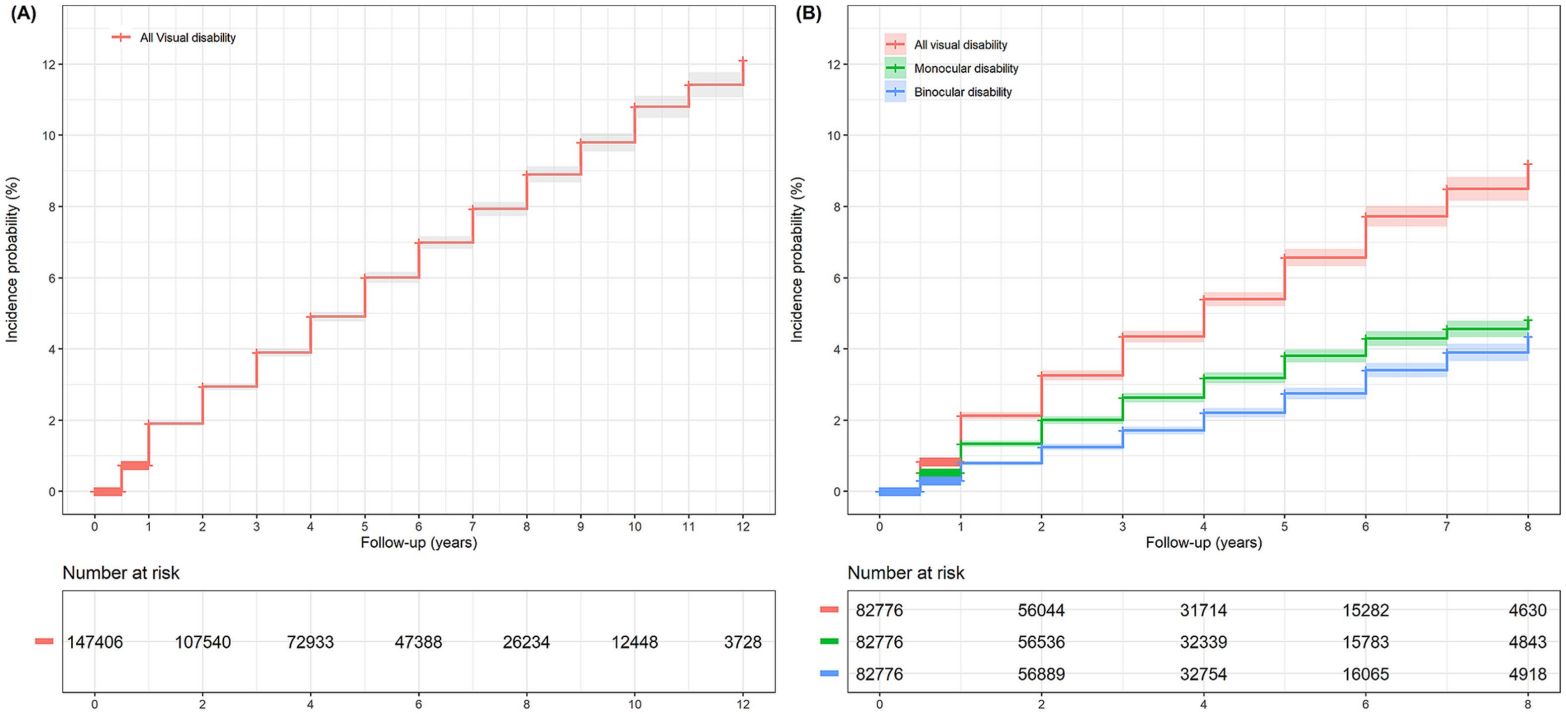
Kaplan–Meier curves showing certification of visual disability in overall **(A)** and monocular and binocular visual disability **(B)** in patients with exudative age-related macular degeneration.

**Figure 3:**
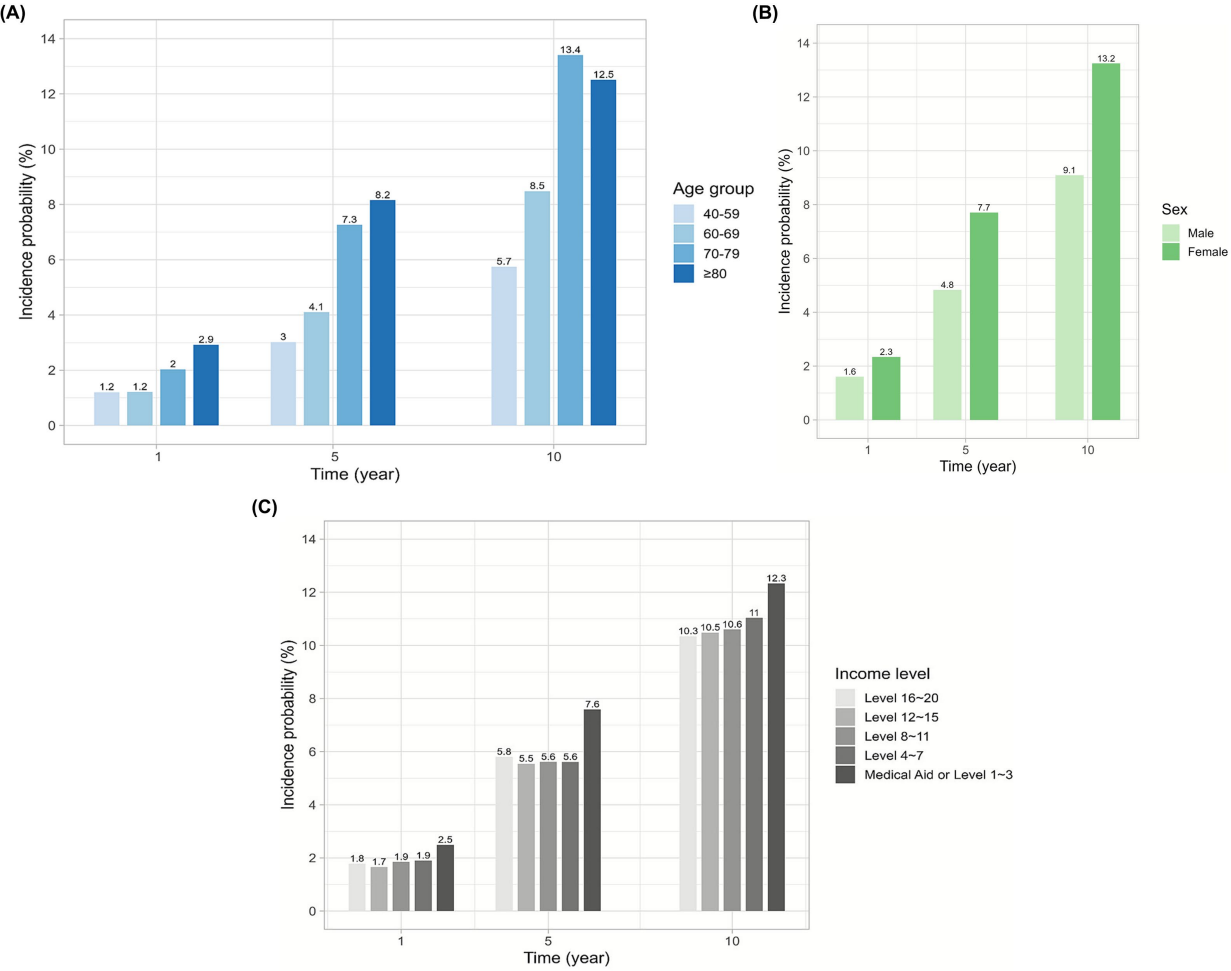
Incidence probability of visual disability at 1-, 5-, and 10-year follow-up by age group **(A)**, sex **(B)**, and income level **(C)**.

**Figure 4:**
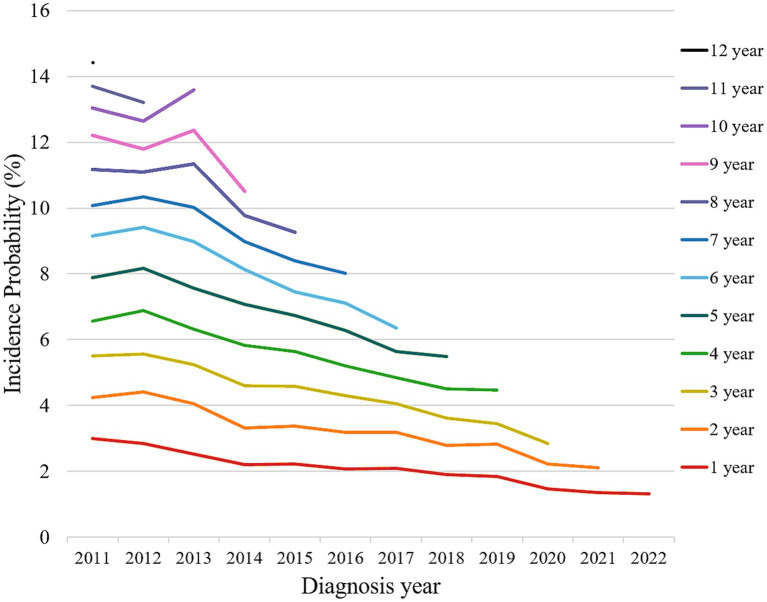
Incidence probability of visual disability stratified by year of exudative age-related macular degeneration diagnosis and follow up years.

In the survival analysis, the cumulative incidence probability of visual disability at 12 years was 12.1% (95% CI, 11.7–12.5) (Figure 2A). In the subgroup analysis, the 8-year incidence probability of monocular visual disability was 4.8% (95% CI, 4.6–5.1), and that of binocular visual disability was 4.4% (95% CI, 4.1–4.7) (Figure 2B). The incidence probability of visual disability at 1, 5, and 10 years showed an increasing trend with older age, female sex, and lower income level (Figures 3A–C). The incidence probability of visual disability showed a decreasing trend among patients who were more recently diagnosed with exudative AMD (Figure 4) (Supplementary Table 4). In the linear mixed-effects model, more recent exudative AMD diagnosis years were associated with a significantly lower annual probability of visual disability during follow-up. Each one-year increase in diagnosis year was associated with a 0.25-percentage point (95% CI, −0.30 to −0.19) decrease in the annual incidence probability of visual disability. In contrast, the risk increased progressively with longer follow-up duration. Compared with the first year after diagnosis, the estimated annual risk rose steadily from year 2 (1.07% point; 95% CI, 0.77–1.37) to year 12 (10.92% point; 95% CI, 10.14–11.69), with all estimates reaching statistical significance (*p* < 0.001) (Supplementary Table 5). The total number of anti-VEGF injections increased over time, from 12,613 in 2011 to 187,169 in 2023. The mean number of injections per patient also rose during the same period, from 2.84 in 2011 to 3.67 in 2023 (Supplementary Table 6).

## Discussion

This nationwide, population-based cohort study investigated the prevalence, incidence and risk factors of visual disability among patients newly diagnosed with exudative AMD in Korea. Using comprehensive claims and visual disability registration data from the National Health Insurance Service, we found that the overall incidence rate of visual disability among patients with exudative AMD was 12.18 per 1,000 person-years, and that 4.8% of prevalent exudative AMD cases had visual disability in 2023.

Our findings extend prior research on the burden of AMD-related visual loss. Globally, AMD remains one of the leading causes of blindness in adults aged 50 years and older, responsible for about 1.8 million cases of blindness and 6.2 million cases of moderate-to-severe vision disability in 2020 (2). AMD accounted for 4.3% of all causes of blindness and 2.1% of moderate-to-severe visual disability worldwide in 2020 (3).

The certification system of visual disability and specific registration code for exudative AMD in Korea provides an opportunity to assess the real-world impact of exudative AMD on functional vision loss rather than solely clinical outcomes. The number of certified patients with visual disability steadily increased over time, reaching its highest level in 2023, with 6,092 cases of visual disability among 126,468 prevalent exudative AMD cases (4.82%). This trend is likely attributable to the growing number of patients living longer with the disease, as well as the increasing incidence and prevalence of exudative AMD, despite the widespread use of anti-VEGF therapy (4, 9, 10).

The increasing rate of visual disability with older age among patients with exudative AMD may be explained by several factors. First, older individuals are more likely to be diagnosed at an advanced stage of the disease. Second, previous studies have shown that older patients tend to exhibit reduced responsiveness to anti-VEGF therapy (11). Third, non-adherence and non-persistence to anti-VEGF treatment are more common in the elderly, which may further contribute to poorer visual outcomes (12). Unfortunately, neither adherence nor response to anti-VEGF therapy cannot be directly assessed using claims data, as it is not possible to determine whether missed injections were due to poor adherence, physician decision-making, or clinical stability.

Another notable finding in our study was that the risk of visual disability increased progressively with lower income levels. Choi et al. (13) reported that all incremental costs in the exudative AMD group were 1.89 to 4.25 and 3.50 to 5.09 times higher in the first and second year, respectively, than those in the control group. Kim et al. (14) also reported that patients with exudative AMD had total costs 2.13 to 4.06 times greater than non-AMD group. These findings suggest that the economic burden associated with anti-VEGF therapy for exudative AMD remains substantial and that individuals with lower income levels are more vulnerable to visual disability.

Exudative AMD is now considered an avoidable cause of visual disability owing to the widespread use of anti-VEGF therapy. The age-standardized prevalence of blindness due to AMD decreased by 11.7% from 2010 to 2019 and by 28% from 1990 to 2020, according to global reports (2). We also found that the patients who were diagnosed with exudative AMD more recently had a lower incidence probability of visual disability during the follow up. This trend may be attributed to the availability of newer anti-VEGF agents such as aflibercept and brolucizumab during the study period, the extended coverage of healthcare insurance, and the increasing adoption of proactive treatment regimens such as the treat-and-extend approach. In our study, we also observed a gradual increase in the mean number of injections per patient over time.

In our study, severe intraocular hemorrhage requiring vitrectomy was identified as a strong risk factor for visual disability, with a hazard ratio of 3.14. Previous studies have shown that 6.7–8% of patients with AMD experience hemorrhagic complications, with an even higher incidence of vitreous hemorrhage reported in the polypoidal choroidal vasculopathy subtype (12.4–19.9%) (15). Large subretinal or submacular hemorrhages can lead to photoreceptor damage and the formation of a disciform scar through mechanisms such as iron toxicity, blockage of nutrient diffusion, and clot retraction (16–18). Consequently, breakthrough vitreous hemorrhage often originates from such large submacular hemorrhages, and despite vitrectomy, visual outcomes are generally poor due to irreversible submacular scarring.

In our study, the incidence rate of visual disability was higher in female, and female sex remained an independent risk factor for visual disability in the Cox regression analysis even after adjusting for age, income level, and comorbidities. Although the underlying mechanisms are not fully understood, potential explanations may include biological susceptibility, differences in disease phenotype, or unmeasured socioeconomic and behavioral factors. Further research incorporating additional clinical and social determinants is warranted to better elucidate the reasons for the higher risk of visual disability observed in female. Cataract was not included in the analysis because claims data lack detailed clinical information on cataract severity, and cataract-related visual disability is relatively uncommon in Korea due to the widespread availability and timely performance of cataract surgery.

### Limitations

This study has several limitations. First, in Korea, certification of visual disability requires patients to visit a medical institution, where an ophthalmologist issues a medical certificate that is subsequently verified by a government agency. Therefore, even if patients meet the criteria for visual disability, the certification process may not be initiated unless they actively request it. As a result, the number of patients with visual disability in this study may have been underestimated, especially in monocular visual disability. Second, the definitions and cut-off values of low vision and blindness vary across countries, making direct comparison between our findings and those of other studies difficult. The World Health Organization classifies visual disability based on Snellen visual acuity, which differ from the criteria used in Korea (19).

## Conclusion

In conclusion, our study provides population-based evidence that 12.18 per 1,000 person-year of patients with exudative AMD in Korea develop visual disability severe enough to warrant official certification. Female sex, older age, lower income, diabetic retinopathy, glaucoma, and intraocular hemorrhage were independent risk factors. These findings highlight the ongoing need for targeted prevention and enhanced support systems for patients at risk of AMD-related visual disability.

## Data Availability

The datasets presented in this article are not readily available because the outflow of source data to the outside is strictly prohibited by national security law. The raw data used in this study can be extracted by request from any qualified investigator. Requests to access the datasets should be directed to Sejoon Woo, sejoon1@snu.ac.kr.
