# Supplementary material for: Prevalence, incidence and risk factors of visual disability in patients with exudative age-related macular degeneration: a nationwide population-based study in Korea
Source: Front Med (Lausanne). 2026 Mar 5;13:1766076. doi: 10.3389/fmed.2026.1766076 (PMC12999404; doi:10.3389/fmed.2026.1766076)
Supplement: Supplementary file 1 [file Supplementary_file_1.docx]

**Supplemental Online Content**

Kim MS, Nam S, Lee J, Woo SJ. Prevalence, Incidence and Risk of Visual Disability in patients with Exudative Age-Related Macular Degeneration: A Nationwide Population-Based Study in Korea. *Frontiers in medicine.*

**eTable 1.** Visual disability classification in Korea

**eTable 2.** Definition of covariates

**eTable 3.** Annual prevalence of visual disability among patients with exudative age-related macular degeneration (AMD)

**eTable 4.** Incidence probability (%) of visual disability stratified by year of exudative age-related macular degeneration diagnosis and follow up years

**eTable 5.** Association of diagnosis year and follow-up year with annual visual disability risk in a linear mixed-effects model

**eTable 6.** Annual trends in the number of anti-VEGF injections and mean injections per patient

This supplemental material has been provided by the authors to give readers additional information about their work.
eTable 1. Visual disability classification in Korea

| Grade | Visual acuity | Visual Field |
| --- | --- | --- |
| 1 | ≤ 0.02 in better eye |  |
| 2 | ≤ 0.04 in better eye |  |
| 3 | ≤ 0.06 in better eye | ≤ 5 degree |
| 4 | ≤ 0.1 in better eye | ≤ 10 degree |
| 5 | ≤ 0.2 in better eye | ≤ 50% |
| 6 | ≤ 0.02 in worse eye |  |

eTable 2. Definition of covariates

| Covariates | Definition | Details |
| --- | --- | --- |
| Hypertension | presence of a hypertension diagnosis code together with antihypertensive medication use | I10-I13, I15; and minimum 1 prescription of anti-hypertensive drug (thiazide, loop diuretics, aldosterone antagonist, alpha-/beta-blocker, calcium-channel blocker, angiotensin-converting enzyme inhibitor, angiotensin II receptor blocker). |
| Diabetes mellitus | presence of a diagnosis code for diabetes together with antidiabetic medication use | E11-E14; and minimum 1 prescription of anti-diabetic drugs (sulfonylureas, metformin, meglitinides, thiazolidinediones, dipeptidyl peptidase-4 inhibitors, α-glucosidase inhibitors, and insulin). |
| Diabetic retinopathy | presence of diabetic retinopathy diagnosis code | H360 |
| Glaucoma | presence of glaucoma diagnosis code | H40, H42 |
| Intraocular hemorrhage requiring vitrectomy | presence of a diagnosis code for intraocular hemorrhage together with a surgical code for vitrectomy | H431 OR H450 OR H356 & S5121, S5122 |

eTable 3. Annual prevalence of visual disability among patients with exudative age-related macular degeneration (AMD)

| Year | Prevalent exudative AMD cases, n | Prevalent visual disability cases, n | Prevalence rate, % (95% CI) |
| --- | --- | --- | --- |
| 2011 | 6372 | 82 | 1.29 (1.01-1.56) |
| 2012 | 12442 | 257 | 2.07 (1.82-2.32) |
| 2013 | 18730 | 493 | 2.63 (2.40-2.86) |
| 2014 | 25321 | 807 | 3.19 (2.97-3.40) |
| 2015 | 34540 | 1191 | 3.45 (3.26-3.64) |
| 2016 | 44484 | 1653 | 3.72 (3.54-3.89) |
| 2017 | 54960 | 2208 | 4.02 (3.85-4.18) |
| 2018 | 66006 | 2838 | 4.30 (4.14-4.45) |
| 2019 | 75191 | 3530 | 4.69 (4.54-4.85) |
| 2020 | 87147 | 4173 | 4.79 (4.65-4.93) |
| 2021 | 99849 | 4819 | 4.83 (4.69-4.96) |
| 2022 | 112730 | 5408 | 4.80 (4.67-4.92) |
| 2023 | 126468 | 6092 | 4.82 (4.70-4.94) |

eTable 4. Incidence probability (%) of visual disability stratified by year of exudative age-related macular degeneration diagnosis and follow up years

|  | 2011 | 2012 | 2013 | 2014 | 2015 | 2016 | 2017 | 2018 | 2019 | 2020 | 2021 | 2022 |
| --- | --- | --- | --- | --- | --- | --- | --- | --- | --- | --- | --- | --- |
| 1 year | 3.0 (2.6-3.4) | 2.8 (2.4-3.3) | 2.5 (2.1-2.9) | 2.2 (1.9-2.5) | 2.2 (1.9-2.5) | 2.1 (1.8-2.3) | 2.1 (1.8-2.4) | 1.9 (1.7-2.1) | 1.9 (1.6-2.1) | 1.5 (1.3-1.7) | 1.4 (1.2-1.5) | 1.3 (1.2-1.5) |
| 2 year | 4.2 (3.7-4.7) | 4.4 (3.9-4.9) | 4.0 (3.6-4.5) | 3.3 (2.9-3.7) | 3.4 (3.0-3.7) | 3.2 (2.8-3.5) | 3.2 (2.9-3.5) | 2.8 (2.5-3.1) | 2.8 (2.5-3.1) | 2.2 (2.0-2.5) | 2.1 (1.9-2.3) |  |
| 3 year | 5.5 (4.9-6.1) | 5.6 (5.0-6.1) | 5.2 (4.7-5.8) | 4.6 (4.1-5.1) | 4.6 (4.2-5.0) | 4.3 (3.9-4.7) | 4.1 (3.7-4.4) | 3.6 (3.3-3.9) | 3.4 (3.1-3.8) | 2.8 (2.6-3.1) |  |  |
| 4 year | 6.6 (5.9-7.2) | 6.9 (6.2-7.5) | 6.3 (5.7-6.9) | 5.8 (5.3-6.4) | 5.6 (5.2-6.1) | 5.2 (4.8-5.6) | 4.8 (4.4-5.2) | 4.5 (4.1-4.9) | 4.5 (4.1-4.9) |  |  |  |
| 5 year | 7.9 (7.2-8.6) | 8.2 (7.5-8.9) | 7.6 (6.9-8.2) | 7.1 (6.5-7.7) | 6.7 (6.2-7.3) | 6.3 (5.8-6.8) | 5.6 (5.2-6.1) | 5.5 (5.1-5.9) |  |  |  |  |
| 6 year | 9.2 (8.4-9.9) | 9.4 (8.7-10.2) | 9.0 (8.3-9.7) | 8.1 (7.5-8.8) | 7.5 (6.9-8.0) | 7.1 (6.6-7.6) | 6.4 (5.9-6.8) |  |  |  |  |  |
| 7 year | 10.1 (9.3-10.8) | 10.4 (9.6-11.1) | 10.0 (9.3-10.8) | 9.0 (8.3-9.7) | 8.4 (7.8-9.0) | 8.0 (7.5-8.5) |  |  |  |  |  |  |
| 8 year | 11.2 (10.4-12.0) | 11.1 (10.3-11.9) | 11.3 (10.5-12.1) | 9.8 (9.1-10.5) | 9.3 (8.7-9.9) |  |  |  |  |  |  |  |
| 9 year | 12.2 (11.4-13.1) | 11.8 (11.0-12.6) | 12.4 (11.5-13.2) | 10.5 (9.8-11.3) |  |  |  |  |  |  |  |  |
| 10 year | 13.0 (12.2-13.9) | 12.7 (11.8-13.5) | 13.6 (12.7-14.5) |  |  |  |  |  |  |  |  |  |
| 11 year | 13.7 (12.8-14.6) | 13.2 (12.3-14.1) |  |  |  |  |  |  |  |  |  |  |
| 12 year | 14.4 (13.4-15.3) |  |  |  |  |  |  |  |  |  |  |  |

eTable 5. Association of diagnosis year and follow-up year with annual visual disability risk in a linear mixed-effects model

| Category | Estimate, % point (95% CI) | *P* |
| --- | --- | --- |
| Diagnosis year | -0.25 (-0.30, -0.19) | <.001 |
| Follow-up  1 year (reference) |  |  |
| 2 year | 1.07 (0.77, 1.37) | <.001 |
| 3 year | 2.09 (1.78, 2.4) | <.001 |
| 4 year | 3.17 (2.85, 3.49) | <.001 |
| 5 year | 4.33 (4.00, 4.67) | <.001 |
| 6 year | 5.43 (5.08, 5.78) | <.001 |
| 7 year | 6.50 (6.12, 6.87) | <.001 |
| 8 year | 7.56 (7.16, 7.96) | <.001 |
| 9 year | 8.57 (8.14, 9.00) | <.001 |
| 10 year | 9.72 (9.23, 10.2) | <.001 |
| 11 year | 10.02 (9.45, 10.59) | <.001 |
| 12 year | 10.92 (10.14, 11.69) | <.001 |

eTable 6. Annual trends in the number of anti-VEGF injections and mean injections per patient

|  | 2011 | 2012 | 2013 | 2014 | 2015 | 2016 | 2017 | 2018 | 2019 | 2020 | 2021 | 2022 | 2023 |
| --- | --- | --- | --- | --- | --- | --- | --- | --- | --- | --- | --- | --- | --- |
| number of anti-VEGF injections | 12613 | 16164 | 21777 | 26691 | 42125 | 48842 | 57742 | 70555 | 84284 | 106983 | 132205 | 158574 | 187169 |
| Injection per patients |  |  |  |  |  |  |  |  |  |  |  |  |  |
| Total | 2.84 | 2.68 | 2.89 | 2.84 | 3.08 | 2.98 | 2.96 | 3.06 | 3.27 | 3.37 | 3.50 | 3.58 | 3.67 |
| 40-59 | 2.83 | 2.62 | 2.79 | 2.64 | 3.04 | 2.91 | 2.93 | 2.91 | 3.03 | 3.14 | 3.19 | 3.30 | 3.37 |
| 60-69 | 2.91 | 2.69 | 2.98 | 2.89 | 3.13 | 3.07 | 3.06 | 3.15 | 3.35 | 3.42 | 3.54 | 3.64 | 3.71 |
| 70-79 | 2.83 | 2.74 | 2.91 | 2.87 | 3.09 | 2.99 | 2.98 | 3.10 | 3.32 | 3.46 | 3.59 | 3.65 | 3.74 |
| ≥80 | 2.71 | 2.58 | 2.77 | 2.79 | 3.01 | 2.87 | 2.83 | 2.94 | 3.16 | 3.22 | 3.42 | 3.50 | 3.61 |

VEGF = vascular endothelial growth factor
